# Gender-Related Differences in Regional Cerebral Glucose Metabolism in Normal Aging Brain

**DOI:** 10.3389/fnagi.2022.809767

**Published:** 2022-02-10

**Authors:** Bei Feng, Jiang Cao, YaPing Yu, HaiYan Yang, YangHongYan Jiang, Ying Liu, Rong Wang, Qian Zhao

**Affiliations:** ^1^Department of Nuclear Medicine, General Hospital of Ningxia Medical University, Yinchuan, China; ^2^Department of Nuclear Medicine, Xi’an Central Hospital, Xi’an, China; ^3^Obstetrics and Gynecology Center Functional Examination Department, General Hospital of Ningxia Medical University, Yinchuan, China

**Keywords:** aging brain, gender differences, 18F-FDG, PET, SPM

## Abstract

**Objectives**: This study was aimed to investigate the gender-related differences of regional cerebral glucose metabolism in healthy people along the age using 18F-FDG PET/CT.

**Methods**: We recruited 344 healthy volunteers, including 217 males and 127 females (age range: 40–89 years old). All subjects underwent fluorine-18 fluorodeoxyglucose(18F-FDG) positron emission tomography (PET). All the data were divided into four groups for every 10 years old. Each participant was carefully screened from PET, MR, and other examinations in order to exclude the abnormalities, such as neurodegenerative or psychiatric disorders, alcohol/abuse, cerebral vascular disorders, metabolic diseases like diabetes mellitus and hyperthyroidism, and other systemic malignancies. The 40–50 years old group was set as the baseline group. Statistical parametric mapping (SPM) analysis was employed to illustrate the differences among groups.

**Results**: Compared to the baseline group, whether in a cohort or different gender groups, the decrease of brain glucose metabolism was shown in the bilateral frontal lobe, anterior cingulate gyrus, and the bilateral temporal lobe. In males, the regions of decreased metabolism were bilateral frontal lobe, caudate nucleus, and cingulate gyrus, whereas that of females were left occipital lobe, cerebellum, and the thalamus. However, the overall decrease of brain metabolism in men and women began from the age of 60s, an aggravated decrease from 70s was only observed in males.

**Conclusion**: (1) An obviously decreased brain metabolism was found from 60 years old, especially in the bilateral frontal lobe, bilateral temporal lobe, and inferior cingulate gyrus; (2) We found specific brain metabolic differences between genders, including the caudate nucleus region in males and the occipital lobe region in females; and (3) The aging trend is different between genders.

## Introduction

Aging can lead to changes in brain function and structure, such as cognitive decline, which indicates dementia, disease, and death (Uchida et al., 2019). A study shows that brain aging may be the initial stage of neurodegeneration (Loewenstein et al., 2004). Another study shows that the pathological changes of familial AD in the brain seem to develop 25 years before clinical symptom onset (Florez, 2012). A robust feature of human biology is that women live longer than men in almost all countries (Austad and Bartke, 2015). In order to detect the preclinical stage of patients of different genders, it is necessary to catch a subtle abnormality that deviates from the healthy state. In other words, it is important to know the healthy brain morphology and activity beforehand, especially between different genders.

Positron emission tomography (Positron Emission Tomography, PET) brain imaging is a functional neuroimaging method that can noninvasively reflect glucose metabolism *in vivo*. Because the change of function is earlier than the structure, PET imaging is more and more widely used in detecting the changes in brain function, such as aging. An early study using FDG-PET showed higher global cerebral glucose metabolic rates in females than in males (Andreason et al., 1994). The latest longitudinal study, which was performed decade-long, showed that functional and morphological changes were affected by gender differences (Thompson et al., 2020). Besides the gender-related differences in brain aging, researches are also focused on the aging speed of the reginal cerebrum. A previous study suggested that some age-related changes in brain structure and metabolism were not linear with age, and showed unequally accelerated changes in the elder people (Brickman et al., 2003).

However, the study on gender-related differences in regional cerebral glucose metabolism in the aging brain has been rarely reported. In this study, we examined the cerebral glucose metabolism using FDG PET/CT in healthy subjects of different genders, and discussed the metabolic differences between sexes and the age-related brain aging.

## Materials and Methods

### Subjects

From November 2014 to December 2018, 344 examinees (age range: 40–89 years) underwent a routine FDG positron emission tomography (FDG PET) in General Hospital of Ningxia Medical University, including 217 males and 127 females. The inclusion criteria were as follows: healthy subjects, age between 40 and 89 years, right-handed, and with complete clinical data. The exclusion criteria were that each participant was carefully screened from PET, MR, and other examinations to exclude the abnormalities, such as neurodegenerative or psychiatric disorders, alcohol abuse, cerebral vascular disorders, metabolic disease like diabetes mellitus and hyperthyroidism, and other systemic diseases. The institutional review board approved the current study. Informed consent was obtained from the subjects after explaining the procedure, risk, and purpose/benefit of the FDG PET study.

### PET Image Analysis

All subjects were asked to fast at least 6 h before scanning. Each of them was injected intravenously with 370 megabecquerel (MBq) of FDG and rested supine with their eyes closed in a quiet, dimly lit room. Imaging was performed with a positron emission tomography scanner (General Electric Company, GE Discovery VCT 64 system). Scanning began 45 min after the injection of FDG. When subjects were positioned in the scanner, a molded headrest and a head restraining Velcro band were applied to firmly secure their heads in order to reduce motion artifact. Whole-body PET images were acquired from the head to upper thighs in the 2-dimensional mode. After finishing the whole-body scan, the brain scan commenced with 4 min 3-dimensional emission scan. The attenuation correction was performed with a uniform attenuation coefficient (μ = 0.096 cm^−1^). In-plane and axial resolution of the scanner was 4.2 and 5.6 mm full width at half maximum (FWHM), respectively.

### SPM Analysis of F-18 FDG Brain PET

In this study, a voxel-by-voxel group analysis was done using SPM8 (Statistical Parametric Mapping 8) running on MATLAB R2014a. The raw data were initially converted from the DICOM to the ANALYZE format using MRIcro (available at www.mricro.com) and transferred to SPM8. MRIcro allows efficient viewing and creation of analyze format headers for exporting brain images to other platforms with common personal computers. After transferring to SPM8, the data were then normalized into the standard PET template provided in SPM8 by using a 12-parameter affine transformation, followed by nonlinear transformations and bilinear interpolation. Dimensions of the resulting voxels were 2 × 2 × 2 mm^3^. Standardized data were then smoothed by a Gaussian filter (full width of half maximum, FWHM = 16 mm). Male and female subjects were analyzed, respectively, with their ages as the covariance to check the relationship between age and brain metabolism. In addition, male subjects were compared with female subjects with age as the nuisance variable to analyze the sex-related differences in brain metabolism. The statistical parametric map SPM was initially obtained at a height threshold T to meet *P* = 0.05 (corrected with familywise error), and then an extent threshold k was set as 100 voxels. The Talairach Daemon database was used to convert the coordinates of these statistically significant areas into corresponding anatomical locations in the Talairach atlas. Results were listed with the Talairach coordinates of the representative peak voxels, as well as their individual k value, t score, and Brodmann area (BA). The k value represents the number of significant voxels in the particular cluster.

## Results

### Subject Characteristics

Table 1 shows the clinical data of healthy subjects. Subjects were divided into four groups by every 10 years old. Each group was compared with reference group (40–50 years old group). We merged the age of 70 and 89 into one group due to the small sample.

**Table 1:** Descriptive statistics of the subjects in this study.

Age groups	Total	Male	Female	Age
(Years old)	(N)	(N, %)	(N, %)	Mean ± Standard
40–49	172	109 (63.4%)	63 (36.6%)	44.65 ± 2.83
50–59	103	72 (69.9%)	31 (30.1%)	53.06 ± 2.87
60–69	44	23 (52.3%)	21 (47.7%)	63.16 ± 3.13
70–89	25	13 (52.0%)	12 (48.0%)	75.25 ± 4.81
Total	344	217 (63.1%)	127 (36.9%)	59.03 ± 3.41

### Changing Pattern of Brain Metabolism Over Ages in Cohort

**Figure 1:**
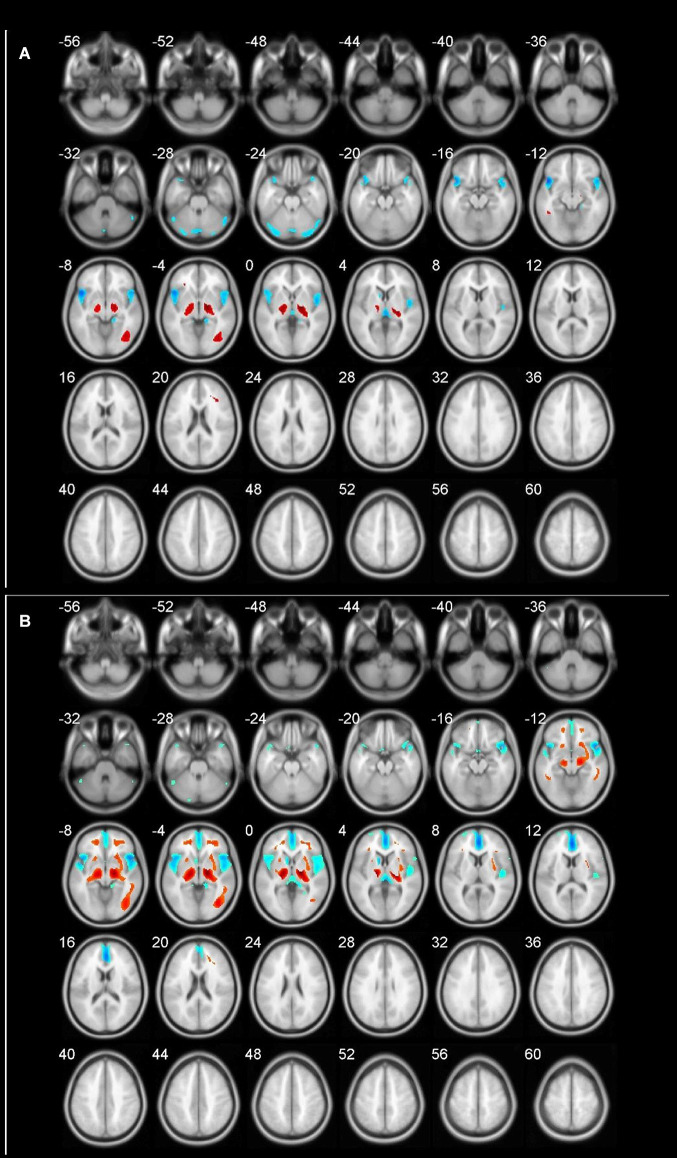
Different brain regions in cohort (blue indicates decrease; red indicates increase). **(A)** 60–69 age group; **(B)** 70–89 age group.

The age-related glucose metabolism differences in cohort were listed in Tables s 2, 3 and Figure 1.

**Table 2:** Brain regions with decreased metabolism in cohort.

Age group (Years old)	Brain regions	*T* value	K_E_ value	Total K_E_	Talairach coordinates			Brodmann
					*x*	*y*	*z*	
50–59	None	None	None	None	-	None	-	None
	Left superior temporal gyrus	6.88	786	2,304	−46	16	−10	38
	Right thalamus	5.87	104		2	−22	6	-
60–69	Right superior temporal gyrus	5.60	762		52	16	−8	38
	Right parahippocampal gyrus	5.06	58		16	−36	−6	30
	Right cerebellum	5.05	265		54	−64	−26	-
	Right anterior cingulate gyrus	10.12	1,718	4,933	2	44	12	32
	Right inferior frontal gyrus	9.00	1,714		48	18	−10	47
	Left inferior frontal gyrus	8.18	922		−44	16	−8	47
70~89	Left caudate nucleus	7.97	105		−10	12	4	-
	Right thalamus	7.60	287		6	−24	6	-
	Left medial frontal gyrus	5.43	40		−2	10	−20	25

*Voxel height threshold *T* = 5.01; *P* = 0.05 with familywise error correction; cluster extent threshold k = 100 voxels*.

**Table 3:** Brain regions with increased metabolism in cohort.

Age group (Years old)	Brain regions	*T* value	K_E_ value	Total K_E_	Talairach coordinates			Brodmann
					*x*	*y*	*z*	
50–59	None	None	None	None	-	None	-	None
	Right lenticular nucleus	6.95	429	1,056	18	−12	−2	-
	Left thalamus	6.35	334		−18	−14	2	-
60–69	Right suboccipital gyrus	5.91	257		38	−72	−4	19
	Right insular lobe	5.58	21		34	24	20	13
	Right lenticular nucleus	10.39	1,364	3,152	18	−12	0	-
	Left thalamus	9.73	730		−16	−12	2	-
	Right suboccipital gyrus	7.38	582		38	−72	−4	19
70–89	Left medial frontal gyrus	5.57	18		−16	48	−6	10
	Right middle frontal gyrus	5.49	296		34	46	−4	11
	Left temporal lobe	5.43	22		−48	−42	−10	37

*Voxel height threshold *T* = 5.01; *P* = 0.05 with familywise error correction; cluster extent threshold *k* = 100 voxels*.

### Changing Pattern of Brain Metabolism Over Ages in Different Gender Groups

**Figure 2:**
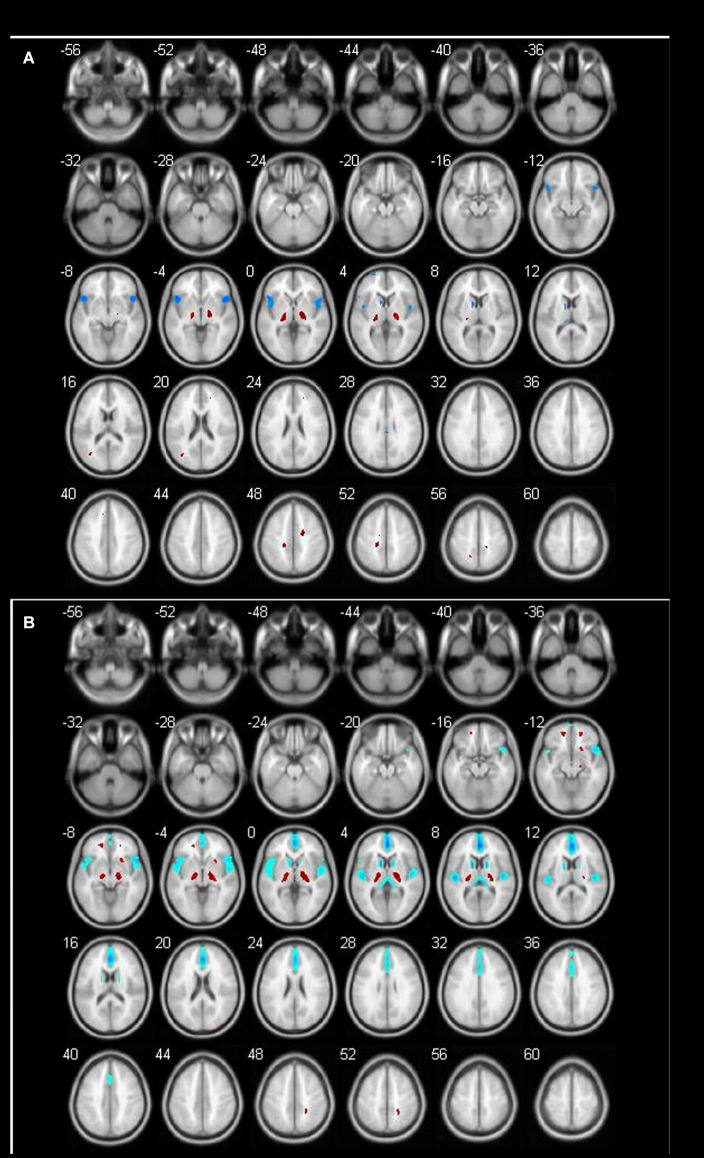
Different brain regions in male groups (blue indicates decrease; red indicates increase). **(A)** Male 60–69 age group; **(B)** male 70–89 age group.

**Figure 3:**
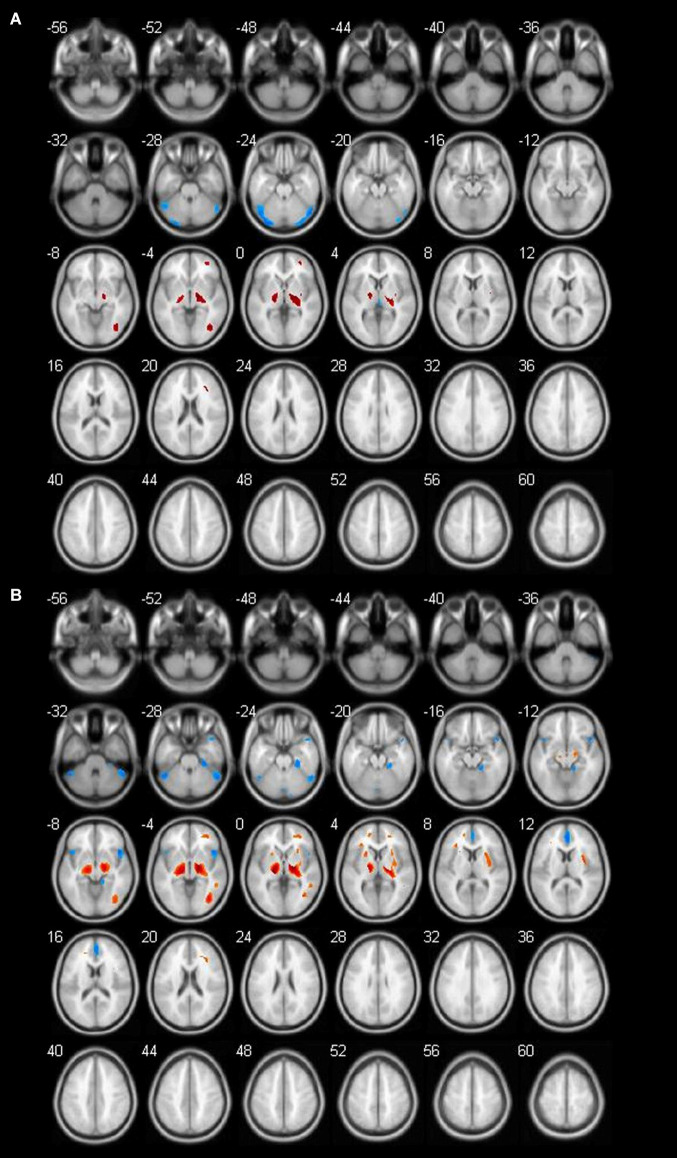
Different brain regions in female groups (blue indicates decrease; red indicates increase). **(A)** Female 60–69 age group; **(B)** female 70–89 age group.

The age-related glucose metabolism differences between males and females were listed in Tables s 4–7 and Figures 2, 3. No decrease or increase metabolic changes were found before the age of 60s in both groups.

**Table 4:** Brain regions with decreased metabolism in males.

Age group (Years old)	Brain regions	*T* value	K_E_ value	Total K_E_	Talairach coordinates			Brodmann
					*x*	*y*	*z*	
50–59	None	None	None	None	-	None	-	None
	Right inferior frontal gyrus	5.60	290	719	50	18	−6	47
	Left inferior frontal gyrus	5.56	333		−46	18	−4	47
60–69	Left caudate nucleus	5.33	40		−10	8	8	-
	Left superior frontal gyrus	5.19	26		−24	66	6	-
	Left thalamus	5.11	13	-	−10	−28	12	-
	Right anterior cingulate gyrus	8.49	2,310	5,027	2	46	6	32
	Left caudate nucleus	7.46	173	-	−12	6	10	-
	Left superior temporal gyrus	7.04	1,097	-	−46	−16	8	22
70–89	Right superior temporal gyrus	6.75	1,056		48	48	8	22
	Right caudate nucleus	6.70	109		14	14	8	-
	Right thalamus	6.41	281		8	8	8	-

*Voxel height threshold *T* = 5.01; *P* = 0.05 with familywise error correction; cluster extent threshold k = 100 voxels*.

**Table 5:** Brain regions with increased metabolism in males.

Age group (Years old)	Brain regions	*T* value	K_E_ value	Total K_E_	Talairach coordinates			Brodmann
					*x*	*y*	*z*	
50–59	None	None	None	None	-	None	-	None
	Right cingulate gyrus	5.88	47	455	18	−8	48	24
	Right lenticular nucleus	5.73	189		18	−12	−2	-
	Left thalamus	5.62	151		−20	−20	2	-
	Right lenticular nucleus	6.81	434	972	18	−12	2	-
	Left thalamus	6.18	287		−18	−14	4	-
70–89	Left anterior cingulate gyrus	5.51	94		−16	46	−8	32
	Right paracenter lobule	5.08	52		22	−44	52	5
	Right claustroid nucleus	5.04	55		26	16	−6	-

*Voxel height threshold T = 5.01; P = 0.05 with familywise error correction; cluster extent threshold k = 100 voxels*.

**Table 6:** Brain regions with decreased metabolism in females.

Age group (Years old)	Brain regions	*T* value	K_E_ value	Total K_E_	Talairach coordinates			Brodmann
					*x*	*y*	*z*	
50–59	None	None	None	None	-	None	-	None
	Left fusiform gyrus	5.71	438	725	−28	−90	−26	18
	Right cerebellum	5.51	257		52	−68	−24	-
60–69	Right thalamus	5.10	30		2	−24	4	-
	Left cerebellum	5.87	173	1,331	−48	−52	−30	-
	Right medial frontal gyrus	5.64	218		2	46	14	10
	Right cerebellum	5.62	307		28	−30	−26	-
70–89	Right inferior frontal gyrus	5.59	273		48	18	−8	47
	Left inferior frontal gyrus	5.17	79		−44	16	−10	47

*Voxel height threshold T = 5.01; P = 0.05 with familywise error correction; cluster extent threshold k = 100 voxels*.

**Table 7:** Brain regions with increased metabolism in females.

Age group (Years old)	Brain regions	*T* value	K_E_ value	Total K_E_	Talairach coordinates			Brodmann
					*x*	*y*	*z*	
50–59	None	None	None	None	-	None	-	None
	Right lenticular nucleus	6.13	324	669	18	−14	−2	-
	Right suboccipital gyrus	6.09	132		40	−74	−6	19
	Right suboccipital gyrus	6.09	132		40	−74	−6	19
60–69	Left thalamus	5.81	148		−18	−14	2	-
	Right insular lobe	5.08	8		32	26	20	13
	Right lenticular nucleus	9.49	937	2,033	18	−12	−2	-
	Left thalamus	9.28	556		−18	−16	0	-
	Right suboccipital gyrus	7.40	194		38	−72	−4	19
70–89	Right middle frontal gyrus	5.68	147		32	48	−2	10
	Left insular lobe	5.66	15		32	26	20	13
	Left inferior frontal gyrus	5.60	35		−30	32	8	47
	Left lenticular nucleus	5.46	52		−22	16	6	-
	Right middle temporal gyrus	5.09	52		52	−48	0	22

*Voxel height threshold *T* = 5.01; *P* = 0.05 with familywise error correction; cluster extent threshold k = 100 voxels*.

### Changing Tendency of Brain Metabolism With Age in Cohort and Different Gender Groups

**Figure 4:**
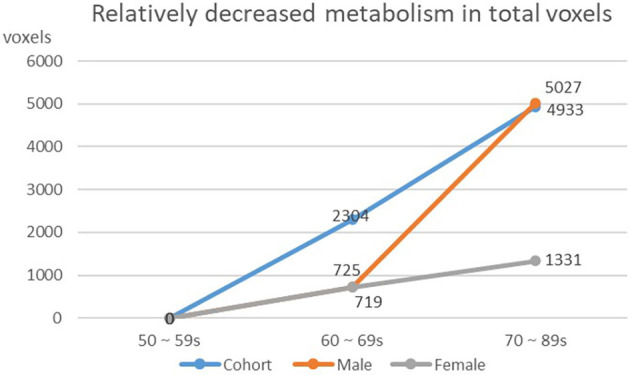
Decreasing of whole brain voxels in cohort and different gender groups.

**Figure 5:**
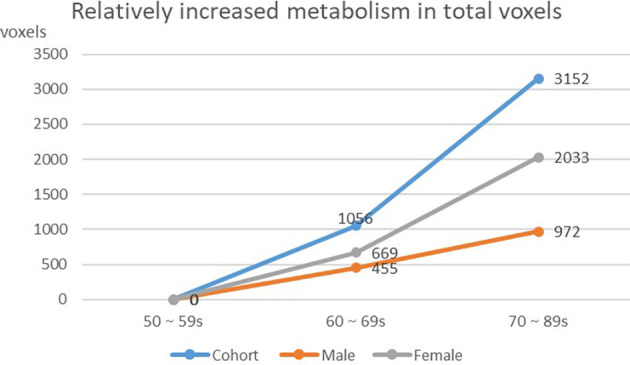
Increasing of whole brain voxels in cohort and different gender groups.

We found that the overall decrease of brain metabolism in men and women were all began from the age of 60s. Interestingly, the trend of decrease was not the same between men and women, i.e., men showed aggravated decrease from the 70s (Table 8, Figures 4, 5).

**Table 8:** Changing of whole brain voxels in cohort and different gender groups.

	Age groups	Relatively decreased metabolism in total voxels			Relatively increased metabolism in total voxels		
		Whole brain	Left brain	Right brain	Whole brain	Left brain	Right brain
Cohort	60–69	2,304	1,114	1,190	1,056	348	708
	70–89	4,933	1,194	3,739	3,152	892	2,260
Male	60–69	719	413	306	455	203	252
	70–89	5,027	1,270	3,757	972	414	558
Female	60–69	725	438	287	669	148	521
	70–89	1,331	280	1,051	2,033	703	1,330

## Discussion

In the past, there were many reports using PET about brain metabolism in neurological, psychiatric, and tumor patients, but little is known about the changes in brain metabolism in aging people of different sexes. Some studies have shown that decreased brain metabolism and brain atrophy mostly occur after the age of 40 (Chance, 2006; Shen et al., 2012). This is the reason why we set the subjects in this experiment as healthy people aged 40 and 89 years old. We investigated the changes in brain metabolism with age in healthy people of different genders. The main finding of our study was as follows: (1) an obviously decreased brain metabolism was found from 60 years old, especially in the bilateral frontal lobe, bilateral temporal lobe, inferior cingulate gyrus; (2) we found specific brain metabolic differences between genders, including the caudate nucleus region in males and the occipital lobe region in females; and (3) the aging trend is different between genders. We discussed and compared the results with other researches as follows.

### Consistent Brain Metabolic Changes Among Cohort, Males and Females

An obviously decreased brain metabolism was found in 60-year-olds, especially in the bilateral frontal lobe (BA10, 11, 25, 47), anterior cingulate gyrus (BA32), and the bilateral temporal lobe (BA22, 30, 38). Meanwhile, the areas of increased brain metabolism were the lenticular nucleus and thalamus.

These findings were consistent with previous studies showing that cerebral metabolic activity decreases gradually with normal aging and primarily affects frontal lobes bilaterally (Beheshti and Kim, 2014). The frontal cortex is the most advanced brain region, which is mainly involved in advanced activities such as body movement and language and is also the brain area most affected by age. Brodmann area 10 (BA 10), is the largest frontal brain region that has been shown to be involved in a wide variety of functions including risk and decision making, odor evaluation, reward and conflict, pain, and working memory (Peng et al., 2018). Recent functional studies have demonstrated that left BA47 has been observed to participate not only in language but also in other domains such as working memory and deductive reasoning, while right BA47 was related with affective prosody as reported (Ardila et al., 2017).

The Anterior Cingulate Cortex (ACC) is an anatomically distinct subregion of the ventromedial frontal cortex consisting of the cingulate sulcus and gyrus that lie dorsal to the corpus callosum and ventral to the superior frontal gyrus. It encompasses Broadmann area 24 and adjacent regions (Gasquoine, 2013). Neuropsychological follow-up of bilateral cingulotomy psychosurgical cases suggests a role for ACC in cognition, specifically executive functioning (Yarkoni, 2009).

Besides frontal lobes and ACC, temporal lobes were involved in many aging-related diseases. We found the decrease of temporal lobes was obvious. Only primates have temporal lobes, which are largest in man, accommodating 17% of the cerebral cortex and including areas with auditory, olfactory, vestibular, visual, and linguistic functions (Kiernan, 2012). A study suggested a series of changes across a wide range of proteins in the human temporal lobe that may relate to aging and age-related neurodegenerative disorders (Xu et al., 2016).

Substrates of memory list learning performance reportedly reside in the anterior part of the brain including the cingulate cortex, frontal cortex, and temporal cortex (Nobili et al., 2007). The frontal aging hypothesis (Tisserand and Jolles, 2003) suggests that hypometabolism in anterior regions including the anterior cingulate gyrus and the frontal lobe is related to executive function and attentional performance, which may decline even in the healthy elderly. Our results, coupled with past studies, support the frontal aging hypothesis.

Not only decreased metabolism was observed, increased regions were also seen along with aging. An increased metabolism was found in the gray matter of the cerebellum and thalamus (Bonte et al., 2017). The results were paralleled with our results. Biswal et al. (2010) using a large sample of over 1,000 subjects have shown reduced resting-state activity in aging mainly in the default model network and increased activity in the visual, motor, and the subcortical regions. The difference from our results was probably due to the sample size and population.

Why do some brain areas increase during aging? We guess the reason is “network”. Given the different rates of declines or relative preservations of different brain regions in aging, and large-scale brain networks working in synchrony during both task execution and resting-state (Biswal et al., 2010), it is likely that the regions that are working together affect each other during the aging process. Specifically, a region that declines faster may influence another region during functional interactions on an everyday basis. For example, the bilateral anterior temporal positively influenced the medial parietal, but negatively influenced the basal ganglia. It is consistent with the direction of the spread of age effects (Di et al., 2019). Therefore, would cause the other region to decline or show a compensatory increase of functional activity.

In addition, the thalamus, with its cortical, subcortical, and cerebellar connections, is a critical node in networks supporting cognitive functions known to decline in normal aging, including component processes of memory and executive functions of attention and information processing (Fama and Sullivan, 2015).

### Inconsistent Brain Metabolic Changes Among Cohort, Males, and Females

In our study, the metabolism of the cerebellum decreased obviously in females, but not in males. The cerebellum is an important, but an understudied region in aging research. The cerebellum plays a role in both motor and cognitive behavior (Ferrucci and Priori, 2014). Atrophy of the cerebellar vermis has been reported to occur with human aging and the age-related loss of Purkinje cells affects most severely the anterior superior vermis in parallel with the ethanol-induced Purkinje cell loss.

In this study, the decrease of bilateral frontal lobe metabolism began in the 60-year-old group in men and the 70-year-old group in women. Some scholars believed that this might be caused by a higher alcohol intake in men than women (Rando et al., 2011).

The fusiform gyrus (FG; BA 18) commonly belongs to a part of the temporal lobe and is considered as a key structure for functionally-specialized computations of high-level vision such as face perception, object recognition, and reading (Weiner and Zilles, 2016). In this study, we found the FG showed a significant decrease along with aging. It is the brain area in 60-year-old women with the most significant decrease in metabolism.

In addition, we found that there was a significant decrease in metabolism in the anterior cingulate gyrus in the male 70-year-old group. A study showed increasing age correlated with significant and extended reduction of brain metabolism in the medial frontal cortex and anterior cingulate gyrus in males (Jaatinen and Rintala, 2008). This result was as same as our results. Another study of 130 healthy people aged 21–90 found that glucose metabolism in the anterior cingulate gyrus decreased with age (Moeller et al., 1996). Some studies indicated that there was an age-related metabolic decrease in the anterior cingulate gyrus accompanied by a decline in cognitive function (Pardo et al., 2007).

Another effect of aging is that bilateral thalamic glucose metabolism increases with aging in males but not in females (Murphy et al., 1996). A study shows that the metabolism of males in the left thalamus increased with aging, though the cause of this increase is unclear (Kawachi et al., 2002). The results were paralleled with our results.

### Metabolic Differences Between Males and Females

In the second part, we discussed inconsistent brain metabolic changes among cohort, male and female. In fact, there were metabolic differences between males and females even in the same age group and made sense. We found specific brain metabolic differences in different genders. In the male group, the brain metabolism decreased to varying degrees in the caudate nucleus region in both the 60-year-old group and the 70-year-old group, while in the female group, the specific decreased brain area appeared in the occipital lobe region in the 60-year-old group.

The age-associated increased FDG uptake regions were clearly different in male and female subjects (Kim et al., 2009). Indeed, it is often said that men outperform women in tasks of visuospatial processing and women outperform men in tasks of speech processing (Strelnikov et al., 2009). The previous studies have recognized that males perform better in the visual-spatial domain, whereas females perform better in the verbal domain of cognitive tasks (Hsieh et al., 2012). A more recent functional MRI (fMRI) study also provides evidences of more prominent brain activation in the occipital cortex in males during visual-spatial cognitive tasks (Bell et al., 2006).

### Trend of Metabolic Changes With Aging

There were some differences in the change trend of brain metabolism between men and women in the previous literature (Baxter et al., 1987; Fujimoto et al., 2008).

In our study, we found that the brain aging of men begins at the age of 60 and shows more after the age of 70, while the brain aging of women begins at the age of 60, and the degree of brain aging at the age of 70 is less than that at the age of 60. These data suggest that women may age slower than men. It has been pointed out that this situation may be related to hormone levels (Marrocco and McEwen, 2016). Studies have shown that estrogen has a certain correlation with emotional control, the protective effects of cerebral vessels and neurons (Murphy et al., 1998). Using estrogen replacement therapy can reduce the risk of AD in women (Sherwin, 2002).

There were also some limitations of our study. The main limitation was the sample size. The aging of the human brain is variational, in our study the data was collected from the age of 40 years old, however the change of brain metabolism was unknown before 40 years old. Further research of big sample size was needed including age before 40 and after 80.

## Conclusion

The conclusions of our study were as follows: (1) an obviously decreased brain metabolism was found from 60 years old, especially in the bilateral frontal lobe, bilateral temporal lobe, and inferior cingulate gyrus; (2) we found specific brain metabolic differences between genders, including the caudate nucleus region in male and the occipital lobe region in female; and (3) the aging trend is different between genders.

## Data Availability Statement

The original contributions presented in the study are included in the article, further inquiries can be directed to the corresponding author.

## Ethics Statement

Ethical review and approval was not required for the study on human participants in accordance with the local legislation and institutional requirements. The patients/participants provided their written informed consent to participate in this study.

## Author Contributions

BF and JC initiated the idea for this article and prepared the final copy of the manuscript. JC is responsible for making pictures and tables. YY, HY, YJ, RW, and YL took responsibility for collecting patient’s data. QZ took responsibility for reviewing this article. All authors contributed to the article and approved the submitted version.

## Conflict of Interest

The authors declare that the research was conducted in the absence of any commercial or financial relationships that could be construed as a potential conflict of interest.

## Publisher’s Note

All claims expressed in this article are solely those of the authors and do not necessarily represent those of their affiliated organizations, or those of the publisher, the editors and the reviewers. Any product that may be evaluated in this article, or claim that may be made by its manufacturer, is not guaranteed or endorsed by the publisher.
